# Study on the Performance Enhancement of Recycled Fine Aggregate Through Carbonation with Calcium Source Supplied by Industrial Waste Residue

**DOI:** 10.3390/ma18071589

**Published:** 2025-04-01

**Authors:** Xuan Li, Chuanjiang Tian, Mao Li, Qiwei Zhan, Xinyu Wang, Wanying Dong

**Affiliations:** 1 School of Civil Engineering and Architecture, Jiangsu University of Science and Technology, Zhenjiang 212100, China; 2 No. Nine Engineering Co., Ltd. of CCCC First Highway Engineering Co., Ltd., Guangzhou 511300, China; 3 School of Material Science and Engineering, Southeast University, Nanjing 211189, China

**Keywords:** recycled fine aggregate, carbonation, calcium source, industrial waste residue

## Abstract

With the rapid advancement of urbanization, the reuse of waste concrete has become more and more important. Recycled aggregate inevitably develops microcracks during the crushing process of waste concrete, resulting in undesirable characteristics such as low density and strong water absorption. This study employed an external calcium source combined with wet carbonation to optimize the performance of recycled fine aggregate (RFA). A series of microscopic analytical techniques, including scanning electron microscopy coupled with energy-dispersive spectroscopy (SEM-EDS), X-ray diffraction (XRD), Fourier transform infrared spectroscopy (FTIR), thermogravimetric analysis (TG), and the Brunauer–Emmett–Teller (BET) method, were used to elucidate the underlying mechanisms. The results indicate that calcium-rich leachate can be obtained by soaking alkali residue in 0.3 mol/L acetic acid at a solid-to-liquid ratio of 1:6. When this leachate was further used to soak the aggregate at a solid-to-liquid ratio of 1:2, followed by carbonation in a carbonation chamber, the carbonation effect reached its optimum. Under these conditions, the saturated water absorption of the recycled fine aggregate decreased to 16%, the carbon sequestration efficiency increased by 66.8%, and pores smaller than 50 nm accounted for 62.9% of the total pore volume. Furthermore, a Bacillus strain capable of producing carbonic anhydrase was introduced to enhance the carbonation reaction. The results demonstrated that when Bacillus was added to acetic acid-modified recycled fine aggregate, the saturated water absorption further decreased to 14.6%, while the carbon sequestration efficiency significantly increased to 109.04%. Additionally, pores smaller than 50 nm constitute 79.2% of the total pore volume. These findings suggest that utilizing calcium-containing industrial waste as a calcium source for recycled fine aggregate, followed by carbonation modification, is highly effective. This approach not only improves the performance of recycled aggregates but also promotes the reutilization of industrial waste, contributing to sustainable construction practices.

## 1. Introduction

With the acceleration of urbanization, the growing demand in the construction industry has led to a continuous increase in construction waste. In China, approximately 2.9 billion cubic meters of concrete are consumed annually for engineering and construction purposes. Concrete is a composite material composed of coarse aggregate, fine aggregate, cementitious materials, and water, with aggregates accounting for nearly 55–80% of its total volume [1,2,3]. Therefore, in order to achieve resource conservation and sustainable development in the construction industry, it is essential to recycle waste concrete by crushing it into aggregates for reuse [4,5,6,7]. Although recycled aggregate contributes to the recycling of construction waste and environmental protection, it also exhibits several drawbacks, such as irregular particle shapes, rough surfaces, and the presence of residual hardened cement mortar. This adhered mortar contains inherent pores and cracks formed during the crushing process, resulting in a higher porosity of recycled aggregate, which adversely affects its water absorption properties [8,9,10,11,12]. Long-term studies conducted both domestically and internationally have demonstrated that recycled aggregate obtained from processed waste concrete can serve as a viable alternative to natural aggregate [13,14,15,16,17].

Scholars worldwide have explored various modification and optimization methods for recycled aggregates, with research focusing on physical treatments, chemical treatments, accelerated carbonation, and microbial treatments. In terms of physical treatment, Bayati et al. [18] investigated the effects of heat treatment at different temperatures. They found that heating at 350 °C combined with mechanical treatment significantly improved the physical properties of recycled aggregate, whereas higher temperatures (500 °C and 750 °C) had adverse effects. Regarding chemical treatment, Panghal H. et al. [19] combined chemical treatment with abrasion techniques and observed that surface-modified recycled fine aggregate (RCA) resulted in compressive, flexural, and splitting tensile strength reductions of 8.31%, 10.61%, and 7.90%, respectively, compared to reference concrete. Pan et al. [20] examined the effects of soaking recycled aggregate in acetic acid solutions of different concentrations and durations. Their results showed that after soaking in a 0.2 mol/L acetic acid solution for 24 h, the water absorption rate decreased by 25%, and the apparent density increased to 2699 kg/m^3^. Feng C. et al. [21] suggested that the silicate gel and calcium silicate hydrate formed under carbonation curing conditions using sodium silicate effectively filled the pore structure of the aggregate. Additionally, Alqarni et al. [22] discovered that treating recycled aggregates with cement-silica fume slurry and sodium silicate solution reduced water absorption and improved both the compressive and tensile strength of recycled concrete. Shaban et al. [23] treated recycled aggregates with volcanic ash-based slurries, such as fly ash-cement, fly ash-silica fume, and nano-silica. Their research indicated that F&S slurry significantly enhanced the strength of recycled concrete. Wang et al. [24,25] investigated the effects of crystallization agents on the density, water absorption, and microstructure of recycled aggregates, revealing that this method improved aggregate performance and increased the compressive strength of concrete by 17.54%. Similarly, Spaeth et al. [26] used polydimethylsiloxane (PDMS) and alkyl alkoxysilane (silane) to modify aggregates, demonstrating that polymer treatment significantly reduced both water absorption and the crushing value. In the field of accelerated carbonation, Zhan [27] conducted carbonation treatment on aggregates using 100% CO_2_ at 1 bar pressure, which significantly improved the interfacial bond performance between recycled aggregate and new materials, as well as the microhardness of the interfacial transition zone. Li et al. [28] studied five treatment methods—pressurized carbonation, gas flow carbonation, wet carbonation, nano-silica spraying, and combined treatments—showing that carbonation treatment improved aggregate properties to varying degrees. For microbial treatments, Wu et al. [29,30] employed a bio-deposition method using *Bacillus pseudofirmus* to induce calcium carbonate precipitation for aggregate treatment, resulting in a 10% and 15% reduction in water absorption and crushing value, respectively. Qian et al. [31] demonstrated that modifying aggregates with carbonic anhydrase-producing bacteria reduced water absorption by 21.2% and increased apparent density to 2620 kg/m^3^, with optimal effects achieved at a bacterial concentration of 1.0%.

This study employs industrial waste as a calcium source for the carbonation of recycled fine aggregate (RFA), with Bacillus bacteria introduced to optimize carbonation efficiency. The research investigates the effects of different solid-to-liquid ratios on saturated water absorption, apparent density, microscopic morphology, and carbon sequestration performance of carbonated RFA under ambient temperature and pressure. Additionally, the enhancement effect of microbial treatment on carbonation efficiency is examined. By calculating the saturated water absorption and apparent density of modified RFA, the optimal solid-to-liquid ratio for industrial waste leaching and aggregate soaking is determined. The mineral composition of carbonated RFA was characterized using X-ray diffraction (XRD) and Fourier transform infrared spectroscopy (FTIR) to analyze its primary crystalline phases and functional group characteristics. The microscopic morphology and particle surface structure of RFA were observed through scanning electron microscopy (SEM) combined with energy-dispersive spectroscopy (EDS), which also enables the analysis of elemental distribution across different regions. Furthermore, thermogravimetric analysis (TG) was employed to quantitatively assess the carbon sequestration capacity of RFA, revealing its thermal decomposition behavior and mass loss patterns during the carbon fixation process. This provided experimental evidence for the application of recycled fine aggregate. The innovation of this paper lies in the carbonization modification of recycled fine aggregate RFA by using industrial waste and microorganisms, which provides a new green way for carbonization modification of recycled fine aggregate RFA.

## 2. Materials and Methods

### 2.1. Raw Material

The primary raw materials used in this experiment are recycled fine aggregate (RFA) and alkaline residue (AR). The RFA was obtained by crushing discarded concrete specimens from the laboratory using a jaw crusher, followed by sieving, while the AR was purchased from a construction materials factory in Gongyi, China.

**Figure 1:**
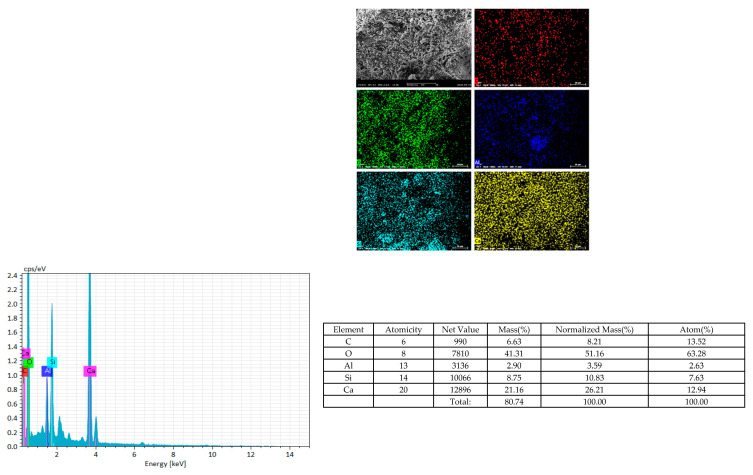
EDS, SEM spectra of RFA.

The chemical composition of both RFA and AR was analyzed using X-ray fluorescence spectroscopy (XRF), and the results are presented in Table 1. As shown in Table 1, RFA primarily consists of SiO_2_, CaO, Al_2_O_3_, and Fe_2_O_3_, with a particularly high SiO_2_ content of 49.4941%. In contrast, AR is mainly composed of CaO, SiO_2_, and MgO, with CaO accounting for as much as 45.2%. Elemental composition analysis of RFA was further conducted using energy-dispersive spectroscopy (EDS), and the results are depicted in Figure 1. As observed in Figure 1, RFA is primarily composed of C, Ca, O, Si, and Al, which is consistent with the findings from the XRF analysis.

The strain used in this experiment was a natural strain named Bacillus M.K., and it was purchased from the China Center of Industrial Culture Collection (Beijing, China). Cultivation of Bacillus M.K. was cultured with shaking in the microbial medium (10 g of sucrose, 3 g of sodium hydrogen phosphate, 0.4 g of magnesium sulfate, 0.5 g of calcium carbonate, 0.1 g of potassium chloride, and 0.4 g of ammonium sulfate were dissolved in deionized water to 1 L, and the pH value was adjusted to about 7.0) at 30 °C for 72 h. A total of 5 g of high-efficiency spore transforming agent manganese chloride was added to the above culture solution, and microbial spore culture solution could be obtained after 6 h. After spray drying, microbial powder was obtained, which was sealed and stored in a dry place for future use.

### 2.2. Experimental Process

First, alkaline residue (AR) was soaked separately in water, acetic acid, and lactic acid at varying concentrations and solid-to-liquid ratios for 24 h. The resulting filtrate was then used to soak recycled fine aggregate (RFA) for another 24 h under different solid-to-liquid ratios. After filtration, the RFA, in a moist state, was placed in a carbonation chamber under controlled conditions: 20% CO_2_ concentration, 80% humidity, and a temperature of 20 °C for 24 h. The carbonated RFA was then removed and dried in an oven. As a control experiment, calcium acetate, calcium lactate, and calcium chloride were used to modify RFA following the same procedure, with different concentrations and solid-to-liquid ratios. The saturated water absorption and apparent density of the modified RFA were measured to determine the optimal concentration and solid-to-liquid ratio for each modification method. Three of the most effective experimental conditions were then selected for further optimization through microbial-enhanced carbonation. Finally, all modified aggregates underwent microscopic analysis and carbon sequestration performance characterization. The specific experimental variable settings are detailed in the Table 2, Table 3, Table 4, Table 5 and Table 6.

### 2.3. Test Method

#### 2.3.1. Mineral Facies Composition

In this experiment, a TD-3500 X-ray diffraction (XRD) instrument (Dandong Tongda Technology Co., Ltd., Dandong, China) was used to analyze the crystalline phase composition of the modified recycled fine aggregate (MRFA). Cu Kα radiation was selected as the X-ray source, and the scanning angle was set between 5° and 85°. To balance testing accuracy with experimental efficiency, the scanning speed was set to 0.3°/s. Since XRD is primarily used to analyze the crystalline phases of metallic and non-metallic materials, it has limited effectiveness in identifying amorphous phases and functional groups within the samples. Therefore, Fourier transform infrared spectroscopy (FTIR-650S) ( Gangdong Technology Co., Ltd., Tianjin, China) was employed as a complementary analysis to obtain chemical functional group information for MRFA. FTIR detects molecular vibration modes by irradiating the sample with infrared light and recording absorption spectra, allowing for the identification of chemical bonds and functional groups present in MRFA. During FTIR testing, the potassium bromide (KBr) pellet method was used to prepare samples, minimizing interference and enhancing signal quality. The experiment was conducted in two stages: first, a background spectrum was collected to eliminate environmental interference; second, the sample spectrum was acquired to obtain its infrared absorption characteristics. A total of 64 scans were performed to ensure the accuracy and reproducibility of the results.

#### 2.3.2. Micro-Morphology and Element Composition

To gain an in-depth understanding of the microscopic morphology of MRFA, this experiment utilized a COXEM EM-30 scanning electron microscope (SEM) (COXEM, Beijing, China) for characterization. Due to the low electrical conductivity of MRFA particles, direct observation may lead to reduced image quality and hinder the identification of microstructural features. Therefore, during sample preparation, a thin layer of gold was uniformly sputtered onto the surface to enhance conductivity, thereby improving imaging resolution and contrast. After sample preparation, the specimens were carefully placed into the SEM chamber, ensuring stable fixation. The chamber was then vacuumed to prevent air interference with the electron beam transmission, ensuring high-quality imaging. The SEM operating parameters, including an accelerating voltage, magnification, and working distance, were optimized to obtain high-resolution microstructural images. During imaging, the surface morphology, particle boundaries, and pore characteristics of MRFA were observed in real-time to analyze its microstructural features and the effectiveness of the modification process. Furthermore, to investigate the elemental composition of MRFA, a QUANTAX XFlash energy-dispersive spectroscopy (EDS) (Bruker, Beijing, China) system was employed. EDS enables qualitative and semi-quantitative analysis of elements within the sample, identifying the primary chemical components and their distribution. During the experiment, spectral scans were conducted on selected areas at different magnifications to obtain comprehensive elemental information, providing critical data for subsequent material performance analysis.

#### 2.3.3. Quantitative Characterization of Carbon Sequestration

Due to the high CaO content in RFA, it possesses a certain potential for carbon sequestration. The theoretical maximum carbon sequestration amount of RFA can be calculated by Equation (1). Based on the contents of CaO, SO_3_, MgO, and K_2_O, the theoretical maximum CO_2_ sequestration capacity (Q_t_) of RFA can be estimated to be approximately 198.01 kg/t [32].

(1) $$Q_{t}=\left[0.785\left(CaO-0.7SO_{3}\right)+1.01MgO+0.93K_{2}O\right]\times 100\%\;$$

In this experiment, an HTG-2 thermogravimetric analyzer (TGA) (Beijing Hengjiu Experimental Equipment Co., Ltd., Beijing, China) was used to determine the carbon sequestration capacity of modified recycled fine aggregate (MRFA). Approximately 12 mg of the sample was weighed and placed in an alumina crucible. The sample was then heated from room temperature to approximately 1000 °C at a heating rate of 20 °C/min. During this process, a precision electronic balance recorded the weight loss of the sample at different temperatures. The carbon sequestration capacity of MRFA was quantitatively calculated using Equation (2) [33,34]. In this equation, W₁ represents the mass loss corresponding to the onset of changes in the differential thermogravimetric (DTG) curve within the temperature range of 550 °C to 850 °C, while W_2_ corresponds to the mass loss at the completion of the changes and the onset of endothermic reactions.

(2) $${CO}_{2uptake}=\frac{W_{1}-W_{2}}{W_{2}}\times 100\%$$

#### 2.3.4. Pore Size Analysis

In this experiment, the pore characteristics were analyzed using a V-Sorb X800 specific surface area and pore size analyzer (Beijing Guoyi precision measurement Technology Co., Ltd., Beijing, China). The specific surface area was determined using the Brunauer–Emmett–Teller (BET) method, while micro-pore analysis was conducted using the Horvath-Kawazoe (HK) method. The measurement range covered specific surface areas as low as 5 × 10^−4^ m^2^/g with no upper limit, while the pore size measurement range spanned 35 nm to 500 nm, and the pore size refers to the diameter. The relative pressure (P/P_0_) control precision ranged from 5 × 10^−6^ to 0.998. Additionally, the pore size distribution of both RFA (Recycled Fine Aggregate) and MRFA (Modified Recycled Fine Aggregate) was measured using a V-Sorb 2800 specific surface area and pore size analyzer (Beijing Guoyi precision measurement Technology Co., Ltd., Beijing, China). During the experiment, approximately 1 g of the sample was placed in a glass tube and subjected to a drying pretreatment before undergoing pore size analysis via the instrument’s operating software (Scientific analysis instrument X800, version: 8,80,860,64228).

#### 2.3.5. Saturated Water Absorption

In this experiment, the quartering method was used to sample 4 kg of recycled fine aggregate (RFA) in its natural state, which was then divided into two portions for backup. The sample was poured into a steel pan, and clean water was added until the water level was approximately 5 mm above the sample surface, with the water temperature maintained at (23 ± 5) °C. A glass rod was used to stir continuously for 5 min to remove excess air bubbles, after which the sample was left to stand for 24 h. After soaking, the water in the steel pan was drained. The sample was then evenly spread in the steel pan, and excess surface moisture was removed to bring the aggregate to a saturated surface-dry (SSD) state. Next, 500 g of the SSD sample (accurate to 0.1 g) was weighed and placed in an oven at (105 ± 5) °C until a constant mass was reached. Once the dried sample cooled to room temperature, its mass was measured again (accurate to 0.1 g). The saturated surface-dry water absorption rate was calculated using the following equation. In the Equation (3), Q is the dry water absorption of the saturated surface, %.m_1_ is the saturated surface dry sample mass, and g.m_0_ is the sample quality after drying, g.

(3) $$Q=\frac{m_{1}-m_{0}}{m_{0}}\times 100\%$$

#### 2.3.6. Apparent Density

In this experiment, approximately 300 g of dried sample was first weighed, ensuring accuracy to 1 g (m_0_). To ensure accurate measurements, the sample must be thoroughly dried under constant temperature conditions to remove any excess moisture. The weighed sample was then slowly poured into a pre-filled volumetric flask containing about half a bottle of clean water. The flask was held and shaken to ensure thorough mixing and complete wetting of the sample. After mixing, the flask’s stopper was tightly secured, and the flask was placed in a constant temperature environment for about 24 h to ensure full contact between the sample and the water, enhancing the reliability of the experiment. After the resting period, clean water was slowly added with a dropper until the water surface was level with the calibration line at the neck of the volumetric flask. During this process, care should be taken to control the water addition rate to avoid overshooting the water level or sample movement that could cause measurement deviation. Once the water level was adjusted, the stopper was securely fastened to ensure good sealing. The outside of the flask was gently wiped with clean, absorbent paper or cloth to remove any residual water that might affect the mass measurement. The total mass of the volumetric flask with the sample was then measured using a balance, ensuring accuracy to 1 g (m_1_). After the initial weighing, the sample and water inside the volumetric flask were carefully poured out. The flask was cleaned repeatedly with clean water to ensure no impurities remained. Once cleaned, clean water was slowly added back into the flask, ensuring the water temperature matched that used in the previous experiment, with a temperature difference not exceeding 2 °C to minimize the effects of thermal expansion or contraction on the measurement results. During the water addition, the flow should be controlled so that the water surface gradually rises to the calibration line at the neck of the flask, and bubbles should be avoided. Once the water level was adjusted, the stopper was replaced, and the outside of the flask was wiped dry to avoid interference from external moisture. Finally, the total mass of the flask was measured again using the balance, ensuring an accuracy of 1 g (m_2_). In the Equation (4), m_0_ is the quantity of dried sample, g; m_1_ is the bottle, sample, and total mass of water, g; m_2_ is the bottle and total water mass, and g; α_t_ is the correction coefficient of water temperature to water relative density. The relative density correction coefficient of water is shown in Table 7.

(4) $$\rho_{as}=(\frac{m_{0}}{m_{0}+m_{2}-m_{1}}-\alpha_{t})\times 1000(kg/m^{3})$$

## 3. Results and Discussion

### 3.1. Macroscopic Performance

**Figure 2:**
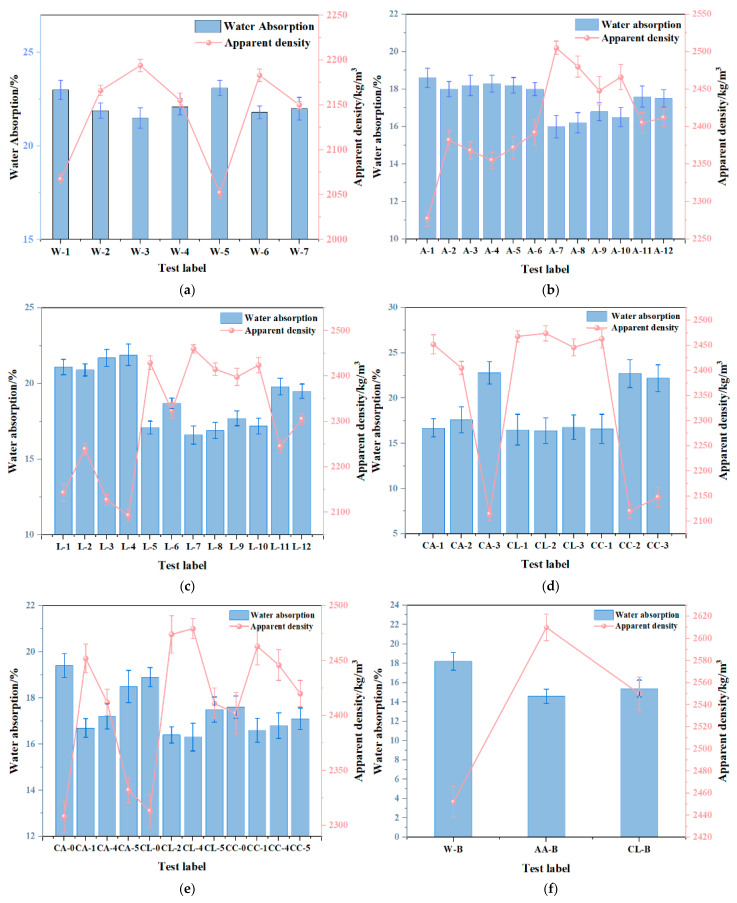
The saturation absorptivity and apparent density (**a**) W (**b**) AA (**c**) LA (**d**) CA, CL, CC (**e**) CA, CL, CC (**f**) W-B, AA-B, CL-B.

Following carbonation, the modified recycled fine aggregate (MRFA) was collected, and its saturated water absorption and apparent density were calculated using Equations (2)–(4). The results indicate that the optimal carbonation effect was achieved under ambient temperature and pressure conditions. It can be seen from the figure that the lower the saturated water absorption rate of the aggregate, the higher its apparent density, and vice versa. It shows that the saturated water absorption of aggregate is inversely proportional to the apparent density. According to the data comparison in the figure, when 0.3 mol/L acetic acid was used to soak the alkaline residue at a solid-to-liquid ratio of 1:6 for 24 h. The resulting filtrate was then used to soak the aggregate at a ratio of 1:2 before carbonation for another 24 h. Under these conditions, the saturated water absorption decreased to 16%, and the apparent density reached 2505 kg/m^3^. Combined with Bacillus carbonization-modified reclaimed fine aggregate, the saturated water absorption decreased to 14.6%, and the apparent density reached 2610 kg/m^3^. The macroscopic performance results of the baseline RFA and the RFA modified through different carbonation methods are presented in the following Figure 2.

### 3.2. Microscopic Characterization

#### 3.2.1. XRD, FTIR

**Figure 3:**
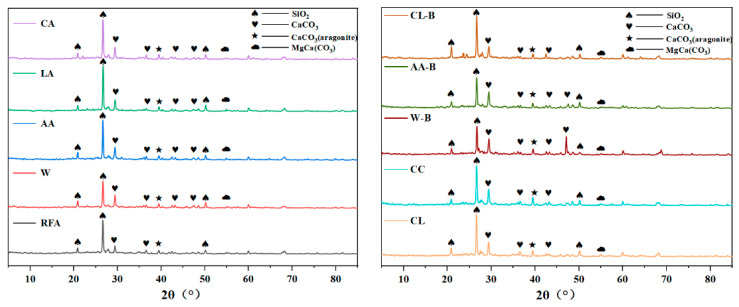
XRD pattern of RHCP with different carbonation pressure.

The XRD patterns of MRFA under different modification methods are shown in Figure 3. As observed, MRFA is primarily composed of silicon dioxide (SiO_2_) and calcium carbonate (CaCO_3_). The diffraction peaks of SiO_2_ mainly appear at 20.86°, 26.62°, and 50.16°. Compared to the diffraction peaks of CaCO_3_, the peak intensity of SiO_2_ is significantly higher, which can be attributed to the high content of siliceous materials in MRFA. These materials exhibit low reactivity and are less likely to participate in chemical reactions, making SiO_2_ the dominant component. The diffraction peaks of CaCO_3_ are primarily located at 29.38°, 36.52°, 39.42°, 43.2°, and 47.5°. The diffraction peak of aragonite is 39.45°. The presence of magnesium calcite (MgCa(CO_3_)_2_) was also found, and its diffraction peak was 54.95°. Notably, in Figure 2, RFA does not exhibit CaCO_3_ diffraction peaks at 43.2° and 47.5°. Similarly, RFA treated with CL and CC does not display CaCO_3_ diffraction peaks at 47.5°, indicating that six modification methods—W, AA, LA, CA, W-B, and AA-B—yield the most effective results.

**Figure 4:**
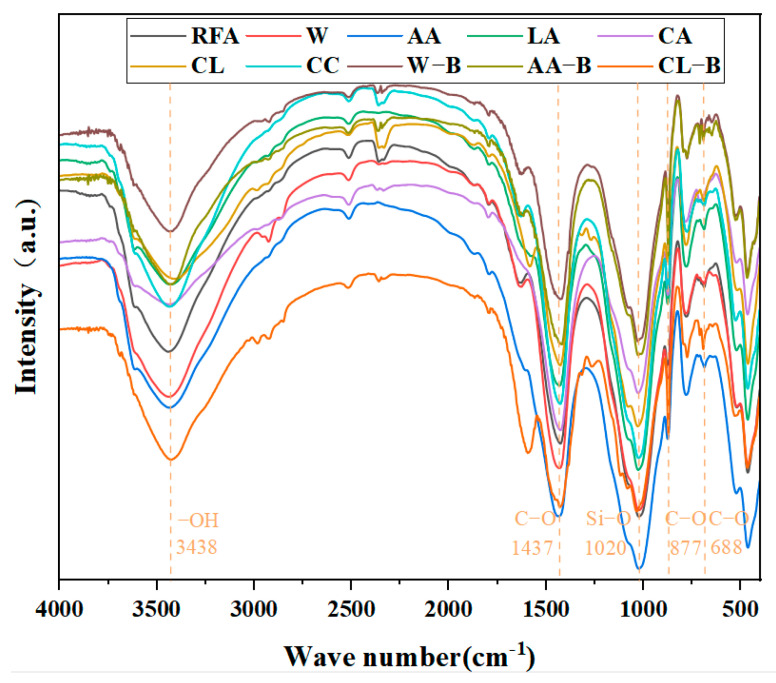
FTIR of RHCP under different carbonation pressures.

The FTIR spectra of MRFA under different modification methods are presented in Figure 4. The spectral curves exhibit similar patterns across all modification methods, suggesting that the modified products are compositionally similar. A distinct and broad vibration peak appears at 3438 cm^−1^, corresponding to the symmetric and asymmetric stretching vibration absorption bands of hydroxyl (-OH) groups in adsorbed water within the product [35]. The diffusion characteristics suggest the presence of either poorly crystalline phases or bound water in MRFA. A vibration peak around 1020 cm^−1^ corresponds to Si-O stretching vibrations [36]. Three prominent vibration peaks at 1437 cm^−1^, 877 cm^−1^, and 688 cm^−1^ correspond to the presence of CaCO_3_ in the product. These peaks represent the asymmetric stretching vibration (v_3_) of C-O, the out-of-plane bending vibration (v_2_) of C-O, and the in-plane bending vibration (v_4_) of C-O, respectively [37,38]. Both XRD and FTIR analyses confirm the formation of CaCO_3_. After the crystal structure is determined by XRD, the chemical bonds are analyzed by FTIR to verify the presence of expected functional groups or chemical bonds in the crystal structure. In XRD images, CaCO_3_ and SiO_2_ showed corresponding characteristic absorption peaks in FTIR images. However, neither method allows for quantitative characterization. Therefore, this study employs thermogravimetric analysis (TG) to quantify the CaCO_3_ content in MRFA, enabling a comparative evaluation of carbonation efficiency across different aggregate modification methods.

#### 3.2.2. TG

**Figure 5:**
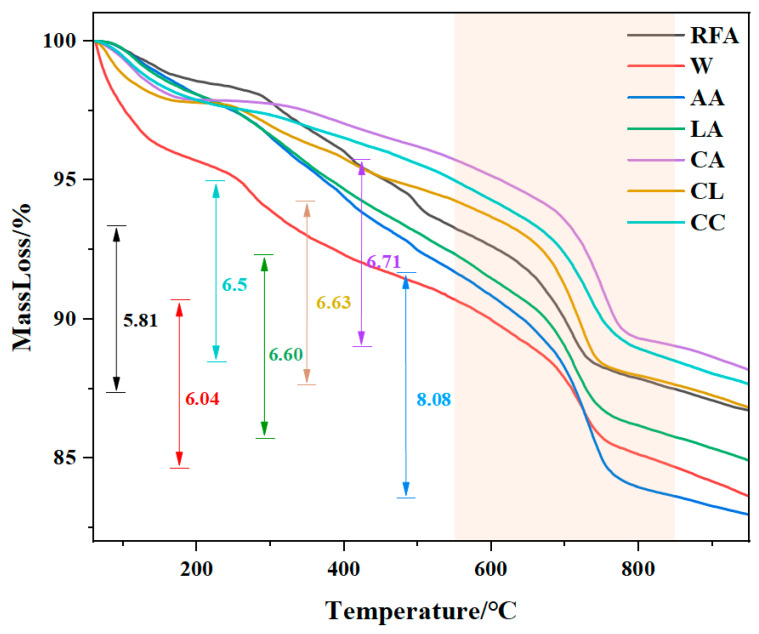
TG of MRFA with different modified modes.

**Figure 6:**
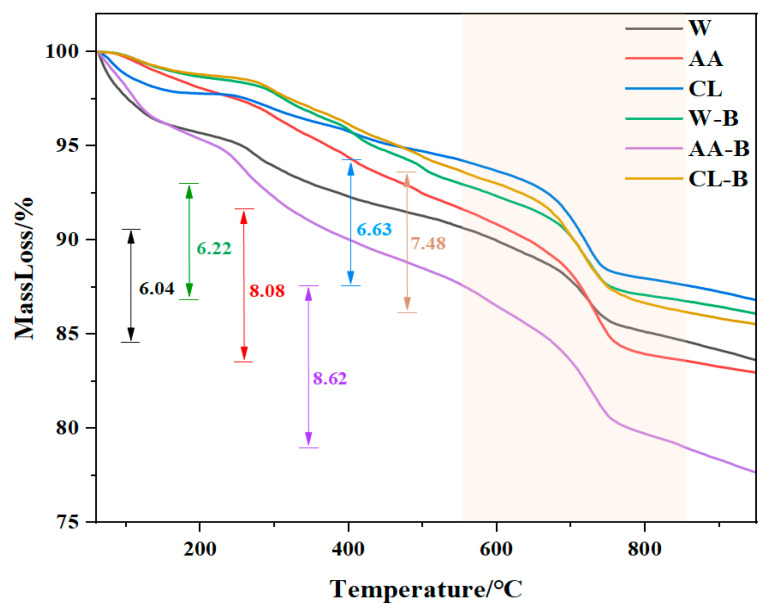
TG of MRFA with Bacillus.

**Figure 7:**
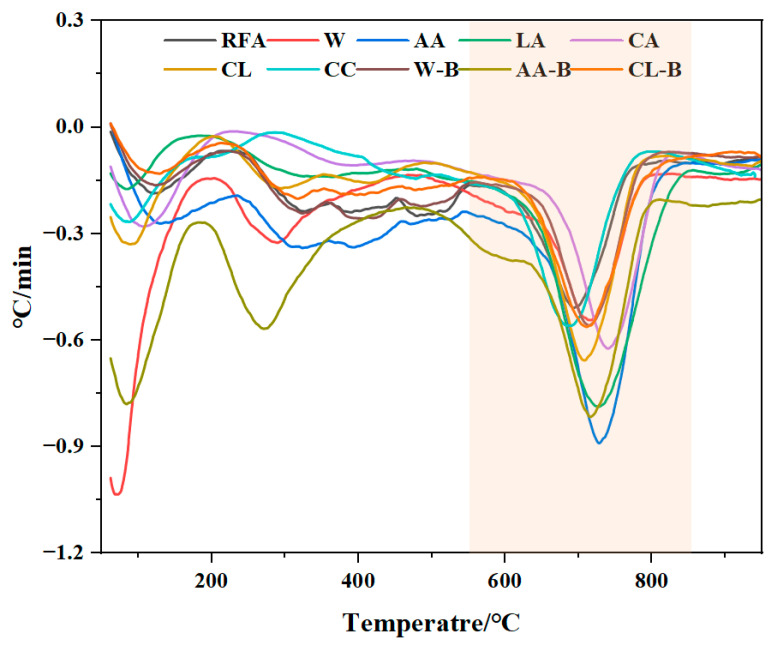
DTG of MRFA.

The TG and DTG analysis results of MRFA under different aggregate modification methods are shown in Figure 5 and Figure 6, respectively. As observed in Figure 7, a distinct mass loss stage occurs between 550 °C and 850 °C, corresponding to the decomposition of CaCO_3_. However, the TG curve alone does not clearly indicate the specific temperature range for CaCO_3_ decomposition, necessitating further analysis using the DTG curve. According to the reviewer’s comments, in thermogravimetric analysis (TG) and differential thermogravimetric analysis (DTG), the peak value of the DTG curve is closely related to the mass change that occurs during the heating process of the material. The temperature corresponding to the DTG peak indicates the point at which the mass change rate is greatest and usually coincides with the inflection point on the TG curve. The peak temperature can be used to identify reaction stages such as decomposition, oxidation, or volatilization of the material at different temperatures. In this study, TG was used to analyze the carbon sequestration amount of modified RFA. According to the relevant literature, the thermal decomposition temperature of calcium carbonate is about 500~900 °C. In combination with the DTG image (Figure 6), it can be seen that a significant decrease interval appears between 550 and 850 °C, indicating that a decomposition reaction of CaCO_3_ occurs in this temperature interval. Therefore, it can be determined that the decomposition temperature of CaCO_3_ generated in this experiment is between 550 °C and 850 °C. Figure 7 presents the DTG curves, which reveal the thermal effects of the samples at different temperatures. A significant endothermic peak appears between 550 °C and 850 °C, confirming that CaCO_3_ decomposition occurs within this temperature range [39]. Since RFA inherently contains a certain amount of fixed CO_2_, this pre-existing carbonation must be accounted for when calculating the carbon sequestration capacity to accurately assess the impact of different modification methods on MRFA. Using Equation (2), the carbon sequestration capacity of different MRFA samples was calculated, with the specific results listed in Table 8. Comparing RFA, W, AA, LA, CA, CL, and CC samples, it was found that AA-treated MRFA exhibited the highest carbon sequestration capacity, increasing by 45.48% compared to unmodified RFA. This indicates that modification using the filtrate obtained from acetic acid-treated alkaline residue significantly enhances MRFA’s carbonation capacity. Further comparison of the W-B, AA-B, and CL-B treatments revealed that the AA-B sample exhibited the highest carbon sequestration capacity, with an increase of 64.17% compared to RFA. This result further validates the effectiveness of the acetic acid-treated alkaline residue filtrate in modifying recycled fine aggregates, highlighting its strong potential for improving MRFA’s carbonation performance.

#### 3.2.3. SEM-EDS

**Figure 8:**
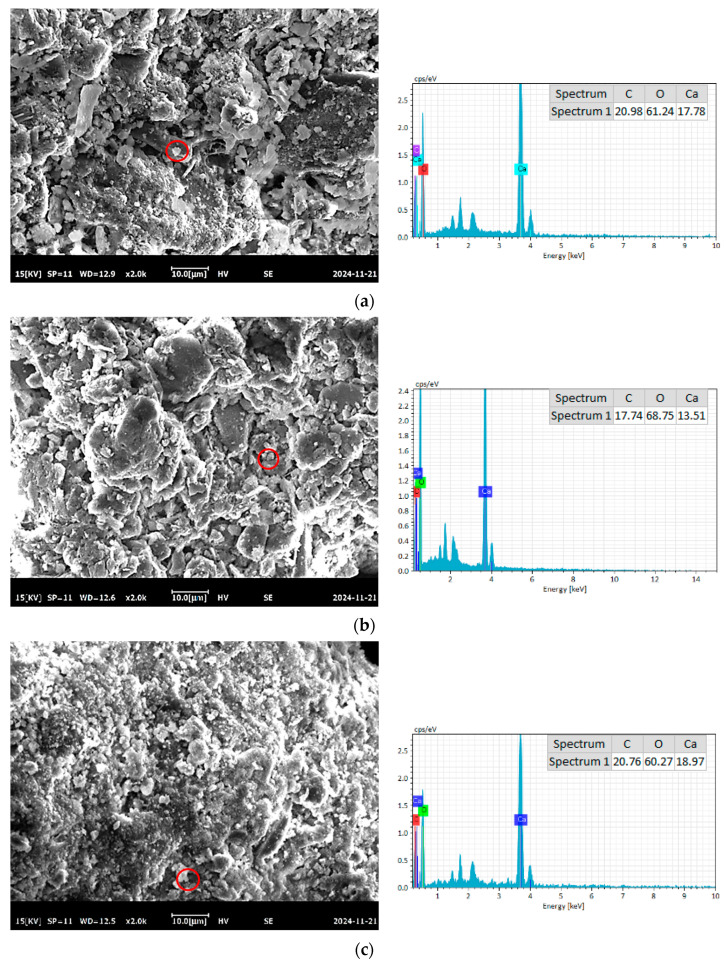

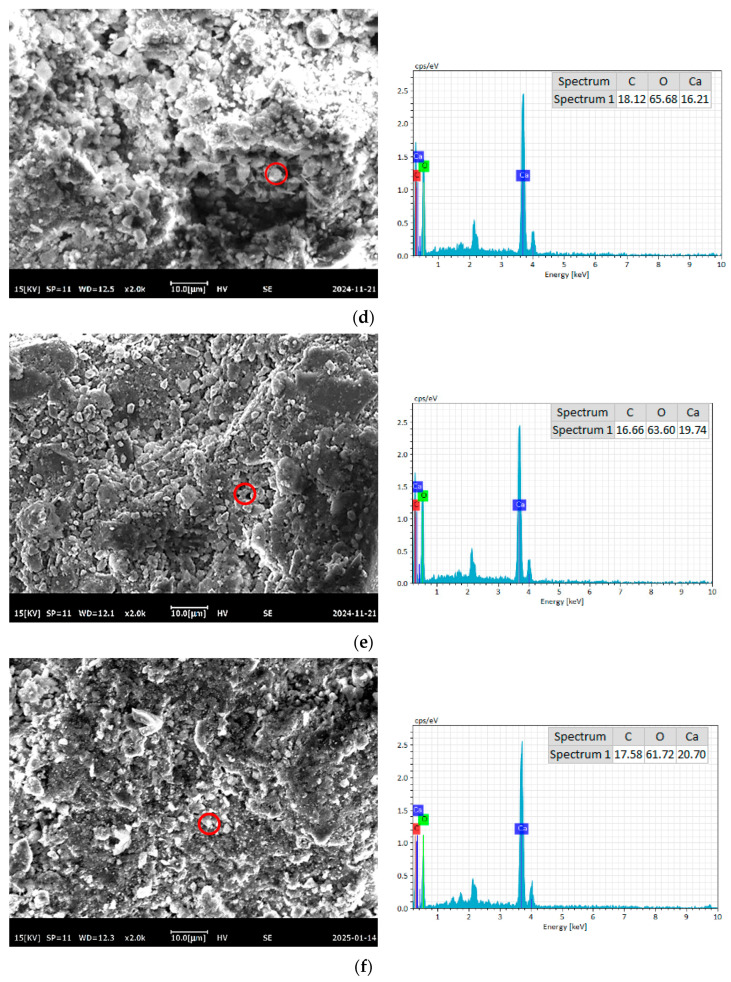

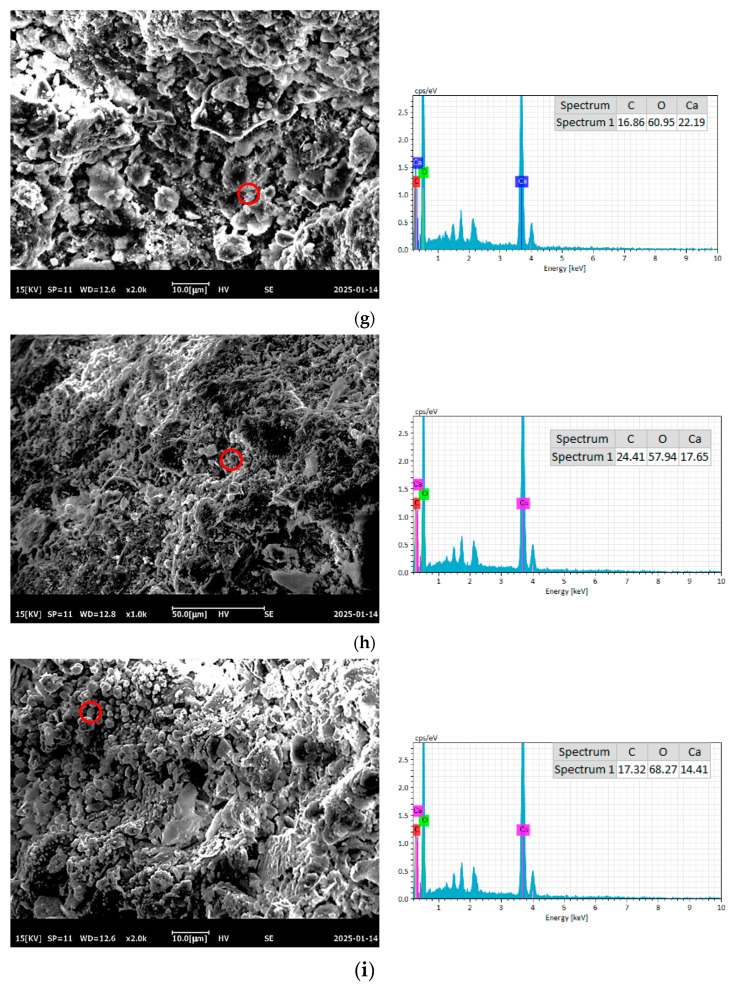
Micro-morphology of MRFA (**a**) W (**b**) AA (**c**) LA (**d**) CA (**e**) CL (**f**) CC (**g**) W-B (**h**) AA-B (**i**) CL-B.

To further compare the differences in recycled fine aggregate (RFA) under different modification methods, scanning electron microscopy (SEM) and energy-dispersive spectroscopy (EDS) were employed to analyze the microstructural morphology of RFA subjected to various carbonation treatments. The test results are presented in Figure 8. As observed in Figure 8a–i, a substantial amount of precipitated reaction products formed on the surface of carbonated RFA, indicating significant changes due to carbonation reactions occurring at the particle surface. EDS scanning of the circle position in Figure 8 was performed to obtain the correlation analysis energy spectrum. The formation of these deposits is closely related to the absorption of CO_2_ and the associated mineral reactions during the carbonation process. After carbonation treatment, numerous precipitates with a well-defined granular structure appeared on the surface of the RFA particles. Elemental analysis using EDS on these precipitated regions revealed that the primary elements present were calcium (Ca), carbon (C), and oxygen (O), confirming that calcium carbonate (CaCO_3_) was the dominant precipitate formed on the RFA surface. The characteristic peaks in the EDS spectrum further substantiate that these deposits are predominantly composed of CaCO_3_. These precipitates not only serve as nucleation sites for hydration reactions but also accelerate the formation of hydration products. This finding aligns with the thermogravimetric analysis (TG) results, which demonstrated an increase in CaCO_3_ deposition during the modification process. The deposited CaCO_3_ exhibits small crystalline particles with a high specific surface area, enabling effective filling of the surface and internal pores of the RFA particles, thereby improving their microstructure. Specifically, fine CaCO_3_ particles fill the internal fine micro-pores of RFA, reducing porosity and enhancing particle density. Additionally, the carbonation-induced deposits may contribute to improved chemical stability of the RFA surface, supporting its long-term hydration performance. The formation of these precipitates not only promotes CaCO_3_ deposition but may also mitigate the adverse effects of surface impurities, facilitating better bonding between RFA and the cement matrix in concrete and ultimately enhancing overall concrete performance. In summary, RFA modification through different carbonation treatments significantly alters its surface characteristics. The deposition of CaCO_3_ effectively enhances the microstructure and long-term stability of RFA, further increasing its potential for use in recycled aggregate concrete. Also, it is expected to enhance the mechanical properties, a fact which will be further investigated in the next article.

#### 3.2.4. BET

**Figure 9:**
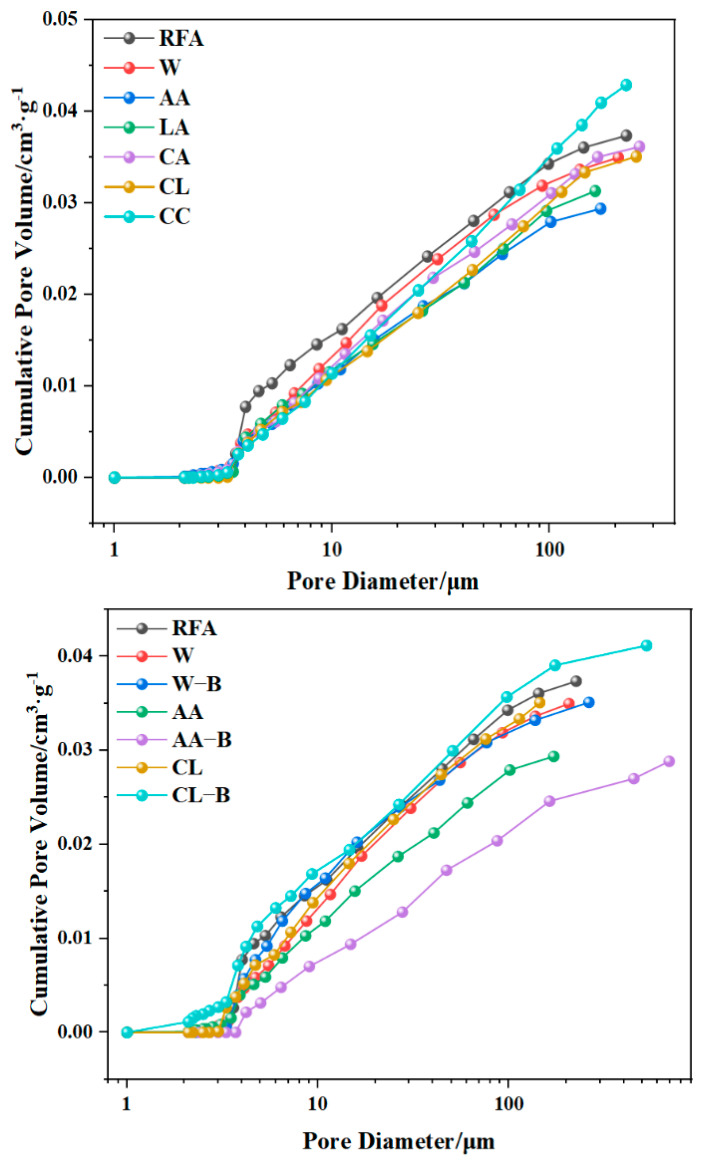
Aperture diagrams of different modification methods.

**Figure 10:**
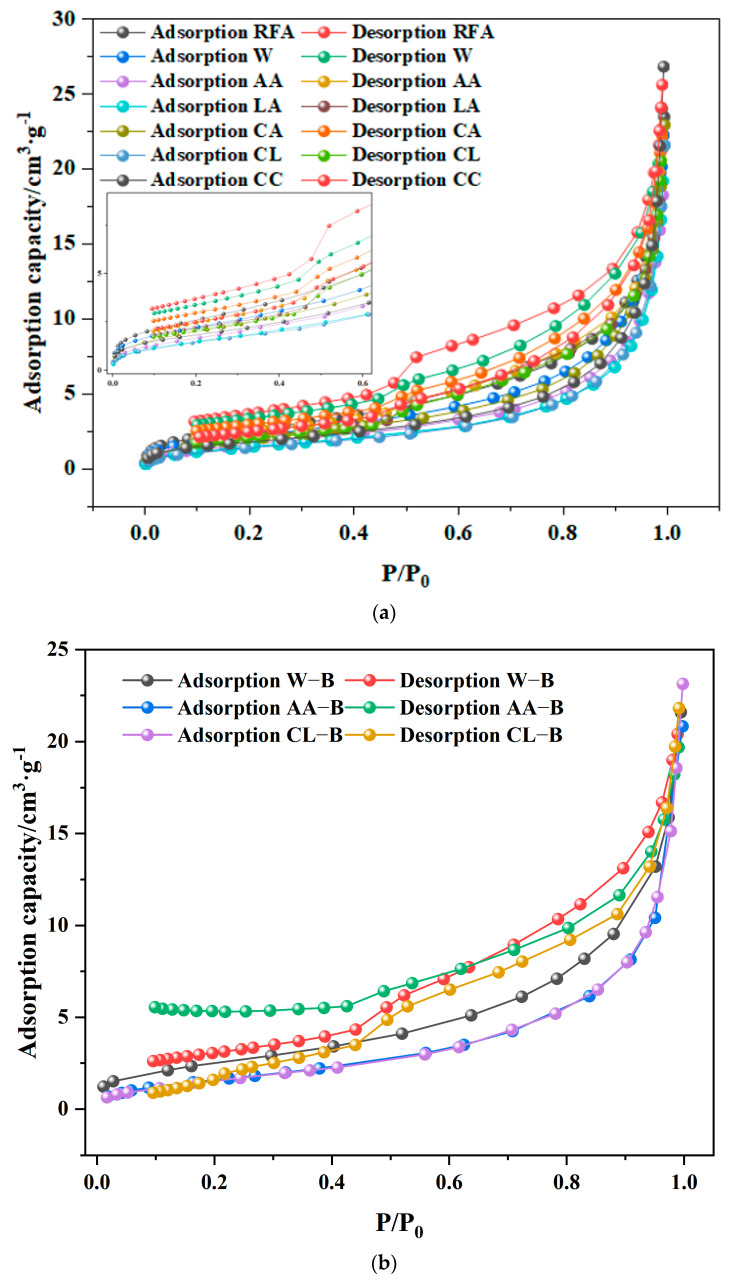
Isotherm diagram of absorption and desorption (**a**) Isotherm diagram of absorption (**b**) Isotherm diagram of desorption.

The pores are classified into three types based on pore size: nano-pores (with a diameter ranging from about 2 nm to 100 nm), submicron pores (with a diameter ranging from 101 nm to 999 nm), fine micro-pores (with a diameter ranging from 1 µm to 10 µm), coarse micro-pores (with a diameter ranging from 10 µm to about 500 µm), and macro-pores (with a diameter ranging from 500 µm to a few millimeters). The pore size distribution of recycled fine aggregate (RFA) under different modification methods is shown in the Figure 9 and Figure 10. It can be observed that both calcium-containing solution carbonation and microbial-induced mineralization treatments predominantly result in pores. The pore distribution is dependent on the modification method applied. It can be seen from the first adsorption pore size diagram that RFA has the largest pore size, and AA and LA have better pore-filling effects. It can be seen from the second adsorption pore size diagram that AA-B has the best filling effect on the pores, which is much better than other methods. These results indicate that the effect of microbial modification and acetic acid modification on fine aggregate regeneration is the best. The cumulative pore volumes of RFA, W, AA, LA, CA, CL, and CC are 3.74 × 10^−2^, 3.50 × 10^−2^, 2.94 × 10^−2^, 3.13 × 10^−2^, 3.62 × 10^−2^, 3.51 × 10^−2^, and 4.29 × 10^−2^ cm^3^/g, respectively. This indicates that the best modification effect on recycled fine aggregate is achieved with the use of acetic-acid-leachate-containing calcium. The cumulative pore volumes for W-B, AA-B, and CL-B are 3.50 × 10^−2^, 2.89 × 10^−2^, and 3.12 × 10^−2^ cm^3^/g, respectively, suggesting that microbial-induced mineralization treatment further improves the mineralization effect. Among these, the combination of acetic acid and bacteria provides the best result, as it exhibits the smallest cumulative pore volume. The proportion of pores smaller than 50 nm in the pore size distributions of RFA, W, AA, LA, CA, CL, and CC are 62.9, 67.3, 77.8, 74.3, 71.7, 77 and 72.3%. For W-B, AA-B, and CL-B, the proportions of pores smaller than 50 nm are 70.1, 79.2, and 79.0%, respectively. This suggests that due to the CO_2_ mineralization reaction in MRFA, new calcium carbonate has filled some pores. Among the treatments, the combination of acetic acid and *Bacillus* sp. provides the best result. The aperture size decreases overall.

### 3.3. Mechanism Analysis

**Figure 11:**
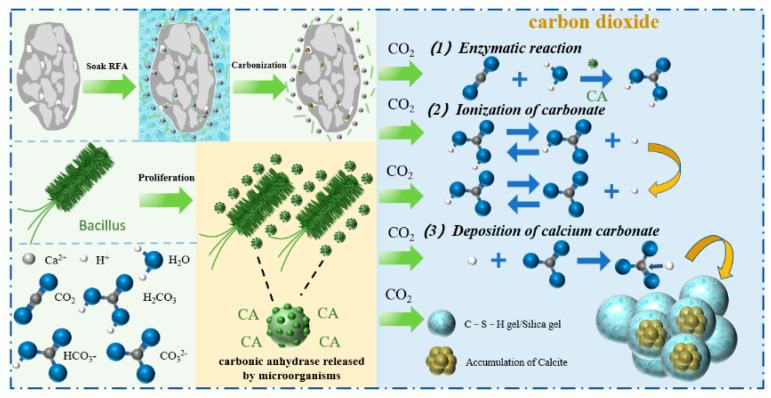
Mechanism analysis diagram.

During the carbonation process, CO_2_ primarily reacted with Ca(OH)_2_ and C-S-H in the mortar adhered to the surface of recycled aggregates. First, CO_2_ penetrated into the adhered mortar of the recycled aggregates through pores or cracks and dissolved in the pore water to form carbonic acid. The Ca^2+^ ions, derived from the decomposition of Ca(OH)_2_, C-S-H, and unhydrated cement clinker minerals, along with Ca^2+^ ions added through the solution, react further with CO_3_^2−^ ions to produce CaCO_3_ and silica gel. The reaction product, calcium carbonate, is a thermally stable and slightly soluble compound, which eventually precipitates in the pores and cracks of the recycled aggregate in the form of calcite, aragonite, or vaterite. As the carbonation reaction progresses, the recycled aggregate carbonates gradually from the outside inward. The internal areas are essentially unchanged because the precipitation of CaCO_3_ hinders the penetration of CO_2_. Since the hardness and solid-phase volume of CaCO_3_ are higher than those of calcium hydroxide and calcium silicate hydrate, the porosity of the recycled aggregate decreases after carbonation, and some of the cracks are filled with reaction products, improving the performance of the recycled aggregate. Furthermore, carbonic anhydrase (CA) in microorganisms can enhance the solubility of carbon dioxide in water [40,41]. Studies have reported that calcium carbonate deposited through microbial methods exhibits small crystal particle sizes and a large specific surface area [42]. A schematic diagram of the mechanism is shown in Figure 11. The upper left of Figure 11 depicts the process of carbonizing RFA, which has a slit on its surface, after being immersed in calcium-containing wastewater and bacterial solution. The bottom left shows how the Bacillus works and the different chemical bonds. On the right is the chemical bond expression for the entire carbonization process.

## 4. Conclusions

This study primarily investigates the carbon sequestration performance and microstructural characterization of recycled fine aggregates (RFAs) under different carbonation modification methods. Based on the experimental results, the following conclusions can be drawn:

(1) Calcium-rich leachate was obtained by immersing alkali residues in 0.3 mol/L acetic acid at a solid-to-liquid ratio of 1:6. This leachate was then used to soak the recycled fine aggregate (RFA) at a solid-to-liquid ratio of 1:2 before undergoing carbonation in a controlled chamber. Under these conditions, the carbonation effect was optimal, reducing the saturated water absorption to 16% and increasing the apparent density to 2505 kg/m^3^.
(2) Comparing six modification methods—W, AA, LA, CA, CL, and CC—Bacillus spores selected by our research group were introduced into the W, AA, and CL treatments. Among them, the AA-B carbonation modification exhibited the most significant improvement, lowering the saturated water absorption to 14.6% and increasing the apparent density to 2610 kg/m^3^. The carbon sequestration capacity was enhanced by 109.04%. These findings indicate that the AA-B carbonation treatment, which combines acetic acid with Bacillus spores, yielded the best macroscopic performance in modifying RFA.
(3) Through XRD image and FTIR image analysis, the changes in regenerated fine aggregate after carbonization modification can be seen from the perspective of microscopic products. The carbonization process produces a small amount of aragonite. Magnesium calcite (MgCa(CO_3_)_2_) was also found in the XRD images due to the use of alkali residue in the experiment, which contains a small amount of MgO.
(4) The microstructural characterization also confirmed the superior performance of AA-B. TG revealed that the carbon sequestration capacity of AA-treated RFA increased by 45.48% compared to untreated RFA, while AA-B treatment further enhanced the carbon sequestration by 64.17%. BET analysis showed that the cumulative pore volume of AA-treated RFA was 2.94 × 10^−2^ cm^3^/g, with pores smaller than 50 nm accounting for 77.8% of the total pore volume. In contrast, AA-B-treated RFA exhibited a slightly lower cumulative pore volume of 2.89 × 10^−2^ cm^3^/g, but the proportion of pores smaller than 50 nm increased to 79.2%. This indicates that incorporating Bacillus spores into the carbonation modification process enhances the overall effectiveness of the treatment.

## Figures and Tables

**Table 1:** The mass percentage of the main chemical composition of RFA.

Material	SiO_2_	CaO	Al_2_O_3_	Fe_2_O_3_	K_2_O	MgO	SO_3_
RFA	49.5	21.9	11.5	7.9	2.4	2.7	1.0
AR	7.3	45.2	1.8	0.5	0	6.6	5.6

**Table 2:** Variable settings of water.

W	AR Solid–Liquid Ratio	RFA Solid–Liquid Ratio
W-1	1:1	1:2
W-2	1:2	1:2
W-3	1:3	1:2
W-4	1:4	1:2
W-5	1:3	1:1
W-6	1:3	1:3
W-7	1:3	1:4

**Table 3:** Variable settings of acetic acid and lactic acid.

Method	Concentration		AR Solid–Liquid Ratio	RFA Solid–Liquid Ratio
AA	0.1	A-1	1:2	1:2
		A-2	1:4	1:2
		A-3	1:6	1:2
		A-4	1:8	1:2
	0.3	A-5	1:2	1:2
		A-6	1:4	1:2
		A-7	1:6	1:2
		A-8	1:8	1:2
	0.5	A-9	1:2	1:2
		A-10	1:4	1:2
		A-11	1:6	1:2
		A-12	1:8	1:2
LA	0.1	L-1	1:2	1:2
		L-2	1:4	1:2
		L-3	1:6	1:2
		L-4	1:8	1:2
	0.3	L-5	1:2	1:2
		L-6	1:4	1:2
		L-7	1:6	1:2
		L-8	1:8	1:2
	0.5	L-9	1:2	1:2
		L-10	1:4	1:2
		L-11	1:6	1:2
		L-12	1:8	1:2

**Table 4:** Variable settings of calcium acetate, calcium lactate, and calcium chloride.

Method	Concentration		RFA Solid–Liquid Ratio
CA	0.1	CA-1	1:2
	0.3	CA-2	1:2
	0.5	CA-3	1:2
CL	0.1	CL-1	1:2
	0.3	CL-2	1:2
	0.5	CL-3	1:2
CC	0.1	CC-1	1:2
	0.3	CC-2	1:2
	0.5	CC-3	1:2

**Table 5:** Variable settings of calcium acetate, calcium lactate, and calcium chloride.

Method	Concentration		RFA Solid–Liquid Ratio
CA	0.1	CA-0	1:1
	0.1	CA-1	1:2
	0.1	CA-4	1:3
	0.1	CA-5	1:4
CL	0.3	CL-0	1:1
	0.3	CL-2	1:2
	0.3	CL-4	1:3
	0.3	CL-5	1:4
CC	0.1	CC-0	1:1
	0.1	CC-1	1:2
	0.1	CC-4	1:3
	0.1	CC-5	1:4

**Table 6:** Variable settings of water, acetic acid, and calcium lactate binding Bacillus.

Method	Concentration	AR Solid–Liquid Ratio	RFA Solid–Liquid Ratio
W-B	\	1:3	1:2
AA-B	0.3	1:6	1:2
CL-B	0.3	\	1:3

**Table 7:** Correction coefficient of relative density of water.

Temperature/°C	17	18	19	20	21	22	23	24
α_t_	0.003	0.004	0.004	0.005	0.005	0.006	0.006	0.007

**Table 8:** Carbon sequestration amount of MRFA under different modification methods.

Methods/MPa	W_1_	W_2_	W_1_-W_2_	CO_2uptake_/g·kg^−1^
RFA	93.29	87.48	5.81	66.42
W	90.71	84.67	6.04	71.34
AA	91.70	83.62	8.08	96.63
LA	92.35	85.75	6.60	77.00
CA	95.74	89.03	6.71	75.37
CL	94.27	87.64	6.63	75.65
CC	94.99	88.49	6.5	73.45
W-B	93.01	86.79	6.22	71.67
AA-B	87.67	79.05	8.62	109.04
CL-B	93.70	86.22	7.48	86.75

## Data Availability

The original contributions presented in this study are included in the article. Further inquiries can be directed to the corresponding authors.
